# Finnish-specific *AKT2* gene variant leads to impaired insulin signalling in myotubes

**DOI:** 10.1530/JME-21-0285

**Published:** 2022-01-04

**Authors:** Selina Mäkinen, Neeta Datta, Savithri Rangarajan, Yen H Nguyen, Vesa M Olkkonen, Aino Latva-Rasku, Pirjo Nuutila, Markku Laakso, Heikki A Koistinen

**Affiliations:** 1 Minerva Foundation Institute for Medical Research, Tukholmankatu, Helsinki, Finland; 2 Department of Medicine, University of Helsinki and Helsinki University Hospital, Haartmaninkatu, Helsinki, Finland; 3 Pam Gene International B.V., Wolvenhoek, BJ ´s-Hertogenbosch, The Netherlands; 4 Department of Anatomy, Faculty of Medicine, Haartmaninkatu, University of Helsinki, Helsinki, Finland; 5 Turku PET Centre, University of Turku, Kiinamyllynkatu, Turku, Finland; 6 Turku PET Centre, Turku University Hospital, Kiinamyllynkatu, Turku, Finland; 7 Institute of Clinical Medicine, Internal Medicine, University of Eastern Finland and Kuopio University Hospital, Puijonlaaksontie, Kuopio, Finland

**Keywords:** *AKT2*, glucose metabolism, insulin signalling, insulin resistance, kinase activity, primary muscle cells

## Abstract

Finnish-specific gene variant p.P50T/*AKT2* (minor allele frequency (MAF) = 1.1%) is associated with insulin resistance and increased predisposition to type 2 diabetes. Here, we have investigated *in vitro* the impact of the gene variant on glucose metabolism and intracellular signalling in human primary skeletal muscle cells, which were established from 14 male p.P50T/*AKT2* variant carriers and 14 controls. Insulin-stimulated glucose uptake and glucose incorporation into glycogen were detected with 2-[1,2-^3^H]-deoxy-D-glucose and D-[^14^C]-glucose, respectively, and the rate of glycolysis was measured with a Seahorse XF^e^96 analyzer. Insulin signalling was investigated with Western blotting. The binding of variant and control AKT2-PH domains to phosphatidylinositol (3,4,5)-trisphosphate (PI(3,4,5)P_3_) was assayed using PIP Strips^TM^ Membranes. Protein tyrosine kinase and serine-threonine kinase assays were performed using the PamGene® kinome profiling system. Insulin-stimulated glucose uptake and glycogen synthesis in myotubes *in vitro* were not significantly affected by the genotype. However, the insulin-stimulated glycolytic rate was impaired in variant myotubes. Western blot analysis showed that insulin-stimulated phosphorylation of AKT-Thr^308^, AS160-Thr^642^ and GSK3β-Ser^9^ was reduced in variant myotubes compared to controls. The binding of variant AKT2-PH domain to PI(3,4,5)P_3_ was reduced as compared to the control protein. PamGene® kinome profiling revealed multiple differentially phosphorylated kinase substrates, e.g. calmodulin, between the genotypes. Further* in silico* upstream kinase analysis predicted a large-scale impairment in activities of kinases participating, for example, in intracellular signal transduction, protein translation and cell cycle events. In conclusion, myotubes from p.P50T/*AKT2* variant carriers show multiple signalling alterations which may contribute to predisposition to insulin resistance and T2D in the carriers of this signalling variant.

## Introduction

Genetic variants in the insulin signalling pathway contribute to increased risk for type 2 diabetes (Manning *et al.* 2012, 2017). One example of an insulin resistance-associated gene is a missense variant p.P50T of *AKT2*, which is specific for Finns (MAF = 1.1%) and very rare in other ancestries. It is associated with higher fasting insulin concentrations and predisposes to type 2 diabetes (Manning *et al.* 2017). The amino acid change from proline (P) to threonine (T) at position 50 lies within the pleckstrin homology (PH) domain of the AKT2 protein. Studies in HeLa cells have demonstrated that this variant leads to a partial loss of AKT2 phosphorylation at its activation sites (Ser^473^/Thr^308^) and to a partial impairment of AKT2’s capacity to phosphorylate its downstream target glycogen synthase kinase 3β (GSK3β) (Manning *et al.* 2017). Analysis of tissue-specific glucose uptake with [^18^F]-fluorodeoxyglucose (FDG)-PET in nondiabetic men with p.P50T/*AKT2* gene variant has revealed that the carriers have reduced insulin-stimulated glucose uptake in multiple tissues including skeletal muscle (Latva-Rasku *et al.* 2018).

AKT is an essential effector in the conserved insulin signalling pathway. The different isoforms of AKT (AKT1, AKT2, AKT3) have tissue-specific expression and distinct functions which include regulation of cell growth, proliferation, survival and metabolic pathways. These are mediated via numerous signalling pathways downstream of AKT (Manning & Cantley 2007, Manning *et al.* 2017). AKT2 is the predominant isoform in human skeletal muscle (Matheny *et al.* 2018) and its inactivating missense mutation leads to severe insulin resistance and type 2 diabetes (George *et al.* 2004). siRNA-based gene silencing of *AKT2* in human primary muscle cells completely blunts insulin action on glucose metabolism, highlighting the importance of this target in the regulation of glucose metabolism in human skeletal muscle (Bouzakri *et al.* 2006).

Given the importance of Finnish-specific p.P50T/*AKT2* gene variant in skeletal muscle glucose metabolism (Latva-Rasku *et al.* 2018), we have established primary muscle cell cultures from the p.P50T/*AKT2* variant carriers and control subjects to study if glucose metabolism and intracellular signalling events are altered *in vitro* in p.P50T/*AKT2* variant carriers.

## Material and methods

The sources of antibodies and reagents are described in the Supplementary Material (see section on supplementary materials given at the end of this article).

### Participants and muscle biopsy

Muscle biopsies were taken with a conchotome under local anesthesia (10 mg/mL lidocaine hydrochloride) from 14 men with p.P50T/*AKT2* gene variant (1 homozygous and 13 heterozygous) and 14 control men (Table 1). These 28 men were recruited from the group of 45 men (20 carriers of p.P50T/*AKT2* gene variant and 25 noncarriers) who participated in the study to measure tissue-specific (including skeletal muscle) glucose uptake during the hyperinsulinemic–euglycemic clamp by the 2-deoxy-2-[^18^F]fluoro-D-glucose-(^18^F-FDG)-PET method (Latva-Rasku *et al.* 2018). HOMA-IR was calculated from fasting plasma glucose and insulin concentrations by the formula: HOMA-IR 
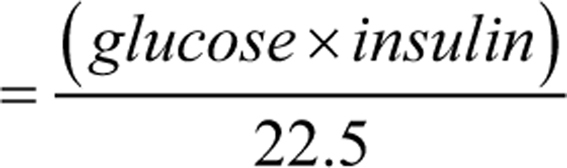
 (Matthews *et al.* 1985, Singh & Saxena 2010). All men are participants in the ongoing Metabolic Syndrome in Men (METSIM)-study (Laakso *et al.* 2017). Muscle biopsies were taken in the fasting state before the start of the hyperinsulinemic–euglycemic clamp. An oral glucose tolerance test was performed to exclude diabetes. The Ethics Committee of the Hospital District of Southwest Finland approved the study protocol. The study was conducted according to the principles of the Declaration of Helsinki, as revised in 2008. All participants gave written informed consent prior to participation in the study.

**Table 1:** Clinical characteristics of all male volunteers

	Controls	p.P50T/*AKT2* carriers	*P*-value
**Whole cohort** (Figs. 1C, D and E)	*n* = 14	*n* = 14	
Age (years)	66.0 ± 0.8	61.4 ± 1.7	0.0220
Weight (kg)	89.7 ± 2.4	85.0 ± 3.0	0.2321
Height (cm)	174.9 ± 1.4	173.3 ± 1.8	0.4818
BMI (kg/m^2^)	29.3 ± 0.7	28.3 ± 1.0	0.3938
Fasting plasma glucose (mmol/L)	6.1 ± 0.1	6.1 ± 0.1	0.9816
Fasting plasma insulin (mU/L)	10.4 ± 1.0	17.9 ± 3.1	0.0281
HOMA-IR	2.8 ± 0.3	4.9 ± 0.9	0.0336
Skeletal muscle glucose uptake (µmol/kg/min)	29.1 ± 3.9	24.4 ± 3.7	0.3839
**Participants in insulin dose–response experiment** (Figs. 1A, B and 2A, B, C, D and E)	*n* = 8	*n* = 10	
Skeletal muscle glucose uptake (µmol/kg/min)	34.0 ± 5.0	20.9 ± 2.2	0.0203
**Participants in kinome profiling experiment** (Figs. 3, 4, 5, 6 and 7)	*n* = 8	*n* = 9	
Skeletal muscle glucose uptake (µmol/kg/min)	32.5 ± 5.6	19.4 ± 2.5	0.0444
**Participants in MNK2 and FAK signalling experiment** (Fig. 8)	*n* = 8	*n* = 9	
Skeletal muscle glucose uptake (µmol/kg/min)	34.0 ± 5.0	21.3 ± 2.4	0.0317

Data are presented as mean ± s.e.m. Statistical analysis was performed using Student´s *t*-test for unpaired data. BMI was not different between the genotypes in any of the experimental subgroups. Skeletal muscle glucose uptake was measured during the hyperinsulinemic–euglycemic clamp by the [^18^F]-FDG-PET method (Latva-Rasku *et al.* 2018).

### Cell culture

Primary muscle cell cultures were established from *vastuslateralis* muscle biopsies (Skrobuk *et al.* 2012, Mäkinen *et al.* 2020). Myoblasts were differentiated into multinucleated myotubes for 6–7 days in low glucose (5.6 mmol/L) DMEM/F12 containing 2% (v/v) FBS. Before the experiments, the myotubes were starved in serum-free, low-glucose DMEM supplemented with 0.5% (w/v) fatty acid-free BSA. All incubations were performed at +37°C, in a 5% CO_2_ incubator. Cell cultures were free of mycoplasma contamination, which was tested frequently.

### Pre-treatment with palmitate

Palmitate conjugated to BSA-NaOH and vehicle control (plain BSA-NaOH) were prepared before each experiment based on the previously reported procedure (Cousin *et al.* 2001). Differentiated myotubes were pre-treated with 0.4 mmol/L palmitate or vehicle control, accompanied by 2 mmol/L l-carnitine, in starvation media for 16–18 h followed by metabolic assays with or without 100 nmol/L insulin.

### Glucose metabolism

Glucose uptake and glucose incorporation into glycogen were measured in differentiated myotubes with radioactive glucose tracers (Skrobuk et al. 2012, Mäkinen et al. 2020) (Supplementary Material). In brief, glucose uptake was detected in triplicate by measuring the intracellular accumulation of 2-[1,2-^3^H]-deoxy-D-glucose (final specific activity 100 mCi/mmol). Cytochalasin B (50 μmol/L) was used to subtract the non-specific glucose uptake. Glycogen synthesis was measured in triplicate by detecting D-[^14^C]glucose (final specific activity 0.18 μCi/μmol) incorporation into glycogen. Radioactivity was measured with a scintillation counter. Values were adjusted to protein concentration measured with a Pierce BCA Protein Assay kit. Data were normalized to the basal control sample of each subject.

Glycolytic rate was determined by measuring extracellular proton efflux rate (PER) with a Seahorse XF^e^96 analyzer (Seahorse Bioscience, a part of Agilent Technologies) using the XF Glycolytic Rate Assay kit (Supplementary Material). Values were adjusted to protein content. Data were normalized to the basal control of each subject.

Intracellular signalling targets were investigated in differentiated myotubes with Western blotting (Skrobuk *et al.* 2012, Mäkinen *et al.* 2020), by detecting the phosphorylated and corresponding total proteins with antibodies, which were visualized by enhanced chemiluminescence and quantified using Fiji software (Schindelin *et al.* 2012) (for signalling targets AKT, AS160 and GSK3β) or Image Lab software (Bio-Rad) (for signalling targets MNK2, eIF4E and FAK) (Supplementary Material). Unless otherwise noted, intensities of the phosphorylated proteins were normalized to the intensity of their corresponding total protein and data were normalized to the basal control sample of each subject.

### Expression and purification of recombinant PH domains of p.P50T/*AKT2* variant and control

Amino acids 1–111 of human PH domain of p.T50/*AKT2* (variant) and p.P50/*AKT2* (control) (NP_001617.1:mnevsvikegwlhkrgeyiktwrpryfllksdgsfigykerpeapdqtl[p/t]plnnfsvaecqlmkterprpntfvirclqwttviertfhvdspdereewmraiqmvanslk) were ordered as pre-cloned to pGEX-4T-1 vector containing the glutathione-S-transferase (GST)-tag to create the C-terminal fusion-partner with AKT-PH domain. Expression vectors were transformed into Rosetta DE3 competent *E. coli* cells for the production of recombinant proteins, which were then purified with Glutathione Sepharose™4B using chromatography columns and eluted with buffer containing 10 mmol/L l-glutathione and 50 mmol/L TRIS pH 8.0.

### PI(3,4,5)P_3_ binding assay

Binding of the p.P50T/*AKT2* and control-form PH domains to phosphatidylinositol (3,4,5)-trisphosphate (PI(3,4,5)P_3_) was assayed using Thermo Fisher PIP Strips™ Membranes according to manufacturer’s protocol. Briefly, protein solution (0.5 μg/mL) was overlayed on BSA-blocked PIP Strips for overnight incubation at 4°C. Bound proteins were probed with GST mouse mAb primary antibody and anti-mouse HRP secondary antibody. The signal was detected with chemiluminescence and visualized with ChemiDoc Imaging System (Bio-Rad). Intensities were quantified with Image Lab 5.1 software (Bio-Rad), by delimiting equal areas of the sample and blank spots on PIP Strips membranes. The signal intensity of the blank spot was subtracted from the signal intensity of the sample spot. Analysis with PIP Strips was repeated four times.

### Cell proliferation assay

The proliferation of non-differentiated myoblasts was determined using WST-1 assay kit (Takara Bio Inc.). Proliferation was followed up to 6 days.

### PamGene® kinome profiling

Comprehensive protein tyrosine kinase (PTK) and serine-threonine (STK) kinase activity assays were performed using the PamGene^®^ kinome array system (PamGene International B.V.,’s-Hertogenbosch, The Netherlands). Briefly, myotubes from nine (one homozygous; eight heterozygous) p.P50T/*AKT2* variant carriers and eight controls were serum-starved for 16–18 h and stimulated with 100 nmol/L insulin for 10 min at 37°C, followed by lysing on ice for 15 min using M-PER Lysis buffer containing 1:100 Halt Protease and Phosphatase Inhibitor Cocktail. Samples were diluted to 5 μg/μL for PTK assay and 1 μg/μL for STK assay, snap-frozen in liquid nitrogen and stored in aliquots at −80°C. Frozen aliquots were thawed on ice and immediately used for the determination of kinase activities.

### PamGene® PTK and STK kinome array

Each PamChip^®^ contains 4 identical porous arrays spotted with distinct 13-amino acid long peptide substrates with phosphosites (196 for the PTK and 144 for STK arrays). Arrays were blocked with 2% (w/v) BSA before an assay mix containing PK buffer, protein lysate (5 μg for PTK and 1 μg for STK assays), ATP (400 μmol/l), the corresponding phospho-specific antibodies (FITC-PY20 for the PTK and untagged Antibody mix for the STK assays) were loaded onto each well. Samples were pumped through the porous arrays to facilitate interaction between the active kinases in the sample and the specific peptide substrates immobilized on the chip. In the PTK assay, images were captured in real time every 5 min for 1 h, while for the STK assay, images were captured after 1 h using a detection antibody. Images were captured using the EVOLVE software across multiple exposure times (ET; 10, 20, 50, 100, 200 ms) and quantified using the BioNavigator software (PamGene®).

### Analysis of PamGene® data

Data analysis was performed using BioNavigator v63 software (BN63; PamGene® International, The Netherlands) integrated with R scripts (Bioconductor R version 3.4.2, 2017, The R Foundation for Statistical Computing). The linear regression slope was calculated using the ET, multiplied by 100 and used as the signal (S100, showing peptide phosphorylation intensity) in comparative analysis. After excluding peptides that were undetectable or without kinetics (PTK) and quality control (QC), 125 out of 196 PTK substrates and 120 out of 144 STK substrates on the PamChip® kinome array were included in the final analysis. Combat correction, a batch correction method, was applied for normalization (Johnson *et al.* 2007), after the Log_2_ transformation of the S100 signals. The ratios of normalized signals of the variant and control samples were used to calculate log fold change for each peptide. For generating the peptide phosphorylation heatmaps and comparing the phosphorylation levels across all the samples, normalized Log_2_ S100 signals were used. Statistical significance was tested using unpaired *t*-tests, and these results were represented by volcano plots generated using BN63. Peptides with a *P*-value < 0.05 were considered a significant change in the degree of phosphorylation of a peptide in the two groups.

### Prediction of upstream kinases

Phosphopeptides that passed the QC were mapped for predicted upstream kinases using the Upstream Kinase Analysis (UKA, version 6) functional scoring tool (PamGene® International), integrated into BN63. Permutation analysis resulted in a specificity score (mapping of peptides to kinases) and a significance score (difference between gene variant carriers and control subjects) for each kinase. Based on the combined scores ((specificity + significance) = median final score), an arbitrary threshold of 1.2 was applied. The median final score was used to rank and predict top kinase hits which were different between the two study groups. The differentially regulated upstream kinases were mapped using an interactive Proteomaps (https://www.proteomaps.net/) (Liebermeister *et al.* 2014). The area size of each polygon represents the median final score in p.P50T/*AKT2* variant carriers compared with the controls.

### Statistical analysis

Data are presented as mean ± s.e.m. Statistical analyses were performed using GraphPad Prism (version 6.0h for Mac OS X). Unless otherwise noted, two-way ANOVA with repeated measurements, followed by Sidak’s *post hoc* test for multiple comparisons was used to analyse data. Unpaired *t*-test was used in PamGene^®^ kinome assay. *P* < 0.05 was considered statistically significant.

## Results

### Participants

Primary muscle cell cultures were established from 14 men carrying p.P50T/*AKT2* gene variant and 14 controls (Table 1). The variant carriers were slightly younger, had higher fasting insulin concentration and higher HOMA-IR. There was no difference in BMI or insulin-stimulated skeletal muscle glucose uptake *in vivo* between the genotypes. However, in experiments where muscle cells from a subset of participants were used, insulin-stimulated skeletal muscle glucose uptake *in vivo* was reduced in variant carriers (Table 1).

### Glucose uptake and glycogen synthesis

**Figure 1:**
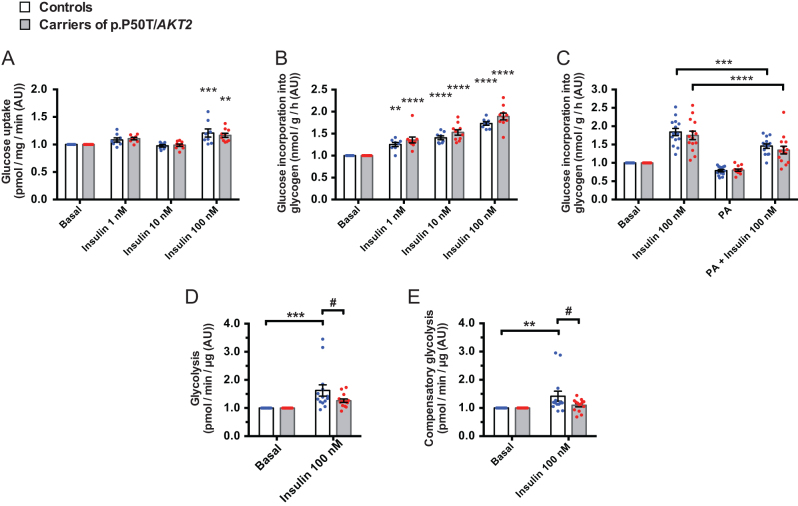
Glucose uptake (A), glucose incorporation into glycogen (B) and the effect of palmitate on glycogen synthesis (C). Primary human myotubes from p.P50T/*AKT2* carriers (*n* = 10) and controls (*n* = 8) were serum-starved for 2 h and stimulated with 0, 1, 10 and 100 nmol/L insulin. Glucose uptake (pmol/mg/min) (A) and glucose incorporation into glycogen (nmol/g/h) (B) were measured using radioactive 2-[1,2-^3^H]deoxy-d-glucose and D-[^14^C]-glucose, respectively. (C) Primary human myotubes from p.P50T/*AKT2* carriers (*n* = 14) and controls (*n* = 14) were serum-starved and pre-exposed to 0.4 mmol/L palmitate (PA) for 16–18 h whereafter glucose incorporation into glycogen in response to stimulation with or w/o 100 nmol/L insulin was measured. Data are expressed as mean ± s.e.m. **P < 0.01, ***P < 0.001 and ****P < 0.0001 vs respective basal, two-way ANOVA with repeated measurements, Sidak’s* post hoc* test. AU, arbitrary units. Glycolysis (D) and compensatory glycolysis (E) were analysed by detecting glycolytic proton efflux rate (PER) in primary human myotubes of p.P50T/*AKT2* carriers (*n* = 14) and controls (*n* = 14) using Seahorse XF^e^96 flux analyzer. Data (in pmol/min/μg) were normalized to protein content and are expressed as mean ± s.e.m. **P < 0.01 and ***P < 0.001 vs respective basal, ^#^*P* < 0.05 vs control subjects, two-way ANOVA, Sidak’s* post hoc* test, AU, arbitrary units. Open bars and blue circles = controls, light grey bars and red circles = carriers of p.P50T/*AKT2*.

Primary human myotubes from p.P50T/*AKT2* variant carriers (*n* = 10) and controls (*n* = 8) were stimulated with increasing concentrations of insulin (1, 10 and 100 nmol/L). Insulin increased glucose uptake (Fig. 1A) and glycogen synthesis (Fig. 1B), with no difference between p.P50T/*AKT2* variant carriers and controls.

We next hypothesized that there might be a gene–environment interaction that accentuates insulin resistance in p.P50T/*AKT2* variant carriers. The saturated fatty acid palmitate (16:0) has been shown to impair insulin-stimulated glycogen synthesis in skeletal muscle cells (Skrobuk *et al.* 2012). In order to examine if variant carriers respond differently to palmitate, we exposed primary human myotubes to 0.4 mmol/L palmitate. Exposure to palmitate led to a significant decrease in insulin-stimulated (100 nmol/L) glycogen synthesis (*P* < 0.001), with no difference between p.P50T/*AKT2* carriers (*n* = 14) and controls (*n* = 14) (Fig. 1C).

### Glycolysis

Baseline glycolytic rate and compensatory glycolysis following mitochondrial inhibition with Antimycin A and Rotenone were measured in primary human myotubes in basal and insulin-stimulated (100 nmol/L) conditions using a Seahorse flux analyzer. Insulin stimulation led to a significant increase in glycolytic rate (*P* = 0.0002) and in compensatory glycolysis (*P* = 0.0051) in control myotubes (*n* = 14), but not in p.P50T/*AKT2* variant myotubes (*n* = 14) (*P* = 0.1626 and *P* = 0.7141 for insulin-stimulated glycolytic rate and compensatory glycolysis, respectively). Comparison between the genotypes revealed a significant reduction in insulin-stimulated glycolysis (Fig. 1D) and compensatory glycolysis (Fig. 1E) in myotubes from p.P50T/*AKT2* variant carriers.

### Activation of the insulin signalling pathway

**Figure 2:**
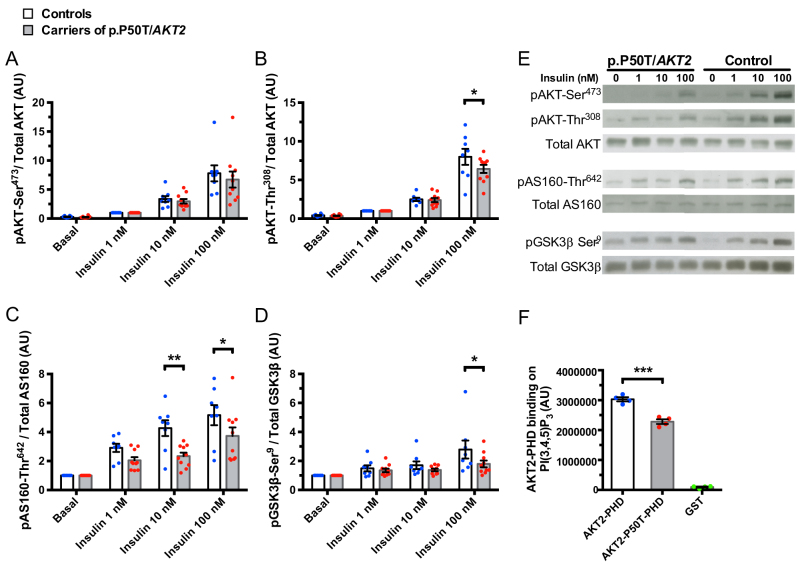
Activation of the insulin signalling pathway. Primary human myotubes from p.P50T/*AKT2* carriers (*n* = 10) and controls (*n* = 8) were serum starved for 2 h and stimulated with 0, 1, 10 and 100 nmol/L insulin, followed by Western blot analysis. Quantification of phosphorylated (p) pAKT-Ser^473^ (A), pAKT-Thr^308^ (B), pAS160-Thr^642^ (C) and pGSK3β-Ser^9^ (D) was normalized to their respective total proteins. Data for pAS160-Thr^642^ and pGSK3β-Ser^9^ were normalized to the basal control sample of each subject. As the pAKT-Ser^473^ and pAKT-Thr^308^ were not reliably detectable at the basal state, the data for pAKTs were normalized to the 1 nM insulin sample of each subject. Representative blots are shown in (E). Data are expressed as mean ± s.e.m. *P < 0.05 and **P < 0.01 vs control subjects, 2-way ANOVA with repeated measurements, Sidak’s* post hoc* test, AU, arbitrary units. (F) Binding of the recombinantly produced variant (p.T50) PH-domain and the wildtype (p.P50) PH-domain of AKT2 to PI(3,4,5)P_3_. Quantification of the PIP Strips™ Membranes was performed by Bio-Rad ImageLab immunoblotting system. Data shown are expressed as average volume intensity from four repeated assays. ***P < 0.001, Student´s unpaired t-test. AU, arbitrary units. Open bars and blue circles = controls, light grey bars and red circles = carriers of p.P50T/*AKT2*, green circles = GST.

Primary human myotubes from p.P50T/*AKT2* variant carriers (*n* = 10) and controls (*n* = 8) were stimulated with increasing concentrations of insulin (1, 10 and 100 nmol/L). Insulin stimulation increased phosphorylation of AKT-Ser^473^, AKT-Thr^308^, AS160-Thr^642^ and GSK3β-Ser^9^ in the myotubes in a dose-dependent fashion (Fig. 2). Insulin-stimulated phosphorylation of AKT-Thr^308^ (Fig. 2B), AS160-Thr^642^ (Fig. 2C) and GSK3β-Ser^9^ (Fig. 2D) was reduced in p.P50T/*AKT2* variant carriers, while there was no statistically significant difference in the phosphorylation of AKT-Ser^473^ (Fig. 2A).

### Binding of the recombinant AKT2-PH domain to PI(3,4,5)P_3_

We recombinantly produced variant and control forms of AKT2-PH domain. The binding of the PH domains to PI(3,4,5)P_3_ was investigated on PIP Strips™ membranes. Binding of the variant form of p.P50T/AKT2-PH domain to PI(3,4,5)P_3_ was reduced (*P* = 0.0004) when compared to the control protein (Fig. 2F).

### Cell proliferation

We observed no difference in cell proliferation between p.P50T/*AKT2* variant carriers and controls (Supplementary Figure 1).

### PTK and STK kinome profiling (PamGene® kinome array system)

**Figure 3:**
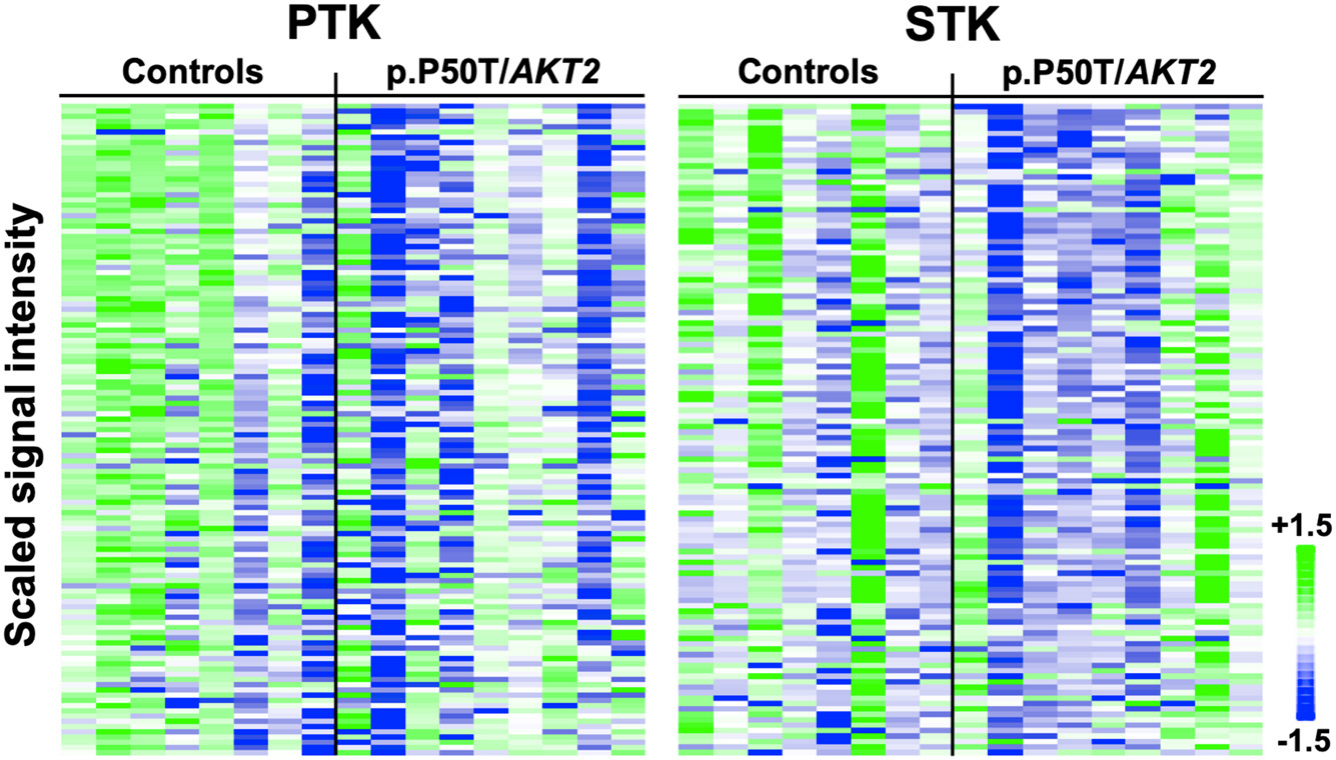
Scaled heatmaps of PTK and STK peptide substrates showing signal intensities of the phosphorylated peptides in arrays exposed to insulin-stimulated muscle cell lysates from p.P50T/*AKT2* variant carriers (*n* = 9) and the controls (*n* = 8). Column represents an array well exposed to insulin-stimulated lysate from a subject (variant carrier or control), and columns are grouped by the genotype. Rows represent the signal intensities of specific phosphorylated peptides, which are scaled per row and sorted according to their correlation with the genotype. Blue colour indicates decreased phosphorylation and green colour indicates increased phosphorylation of a specific peptide, respectively. Scale 1.5 to (−1.5) represents the scaled Log_2_ intensities.

**Figure 4:**
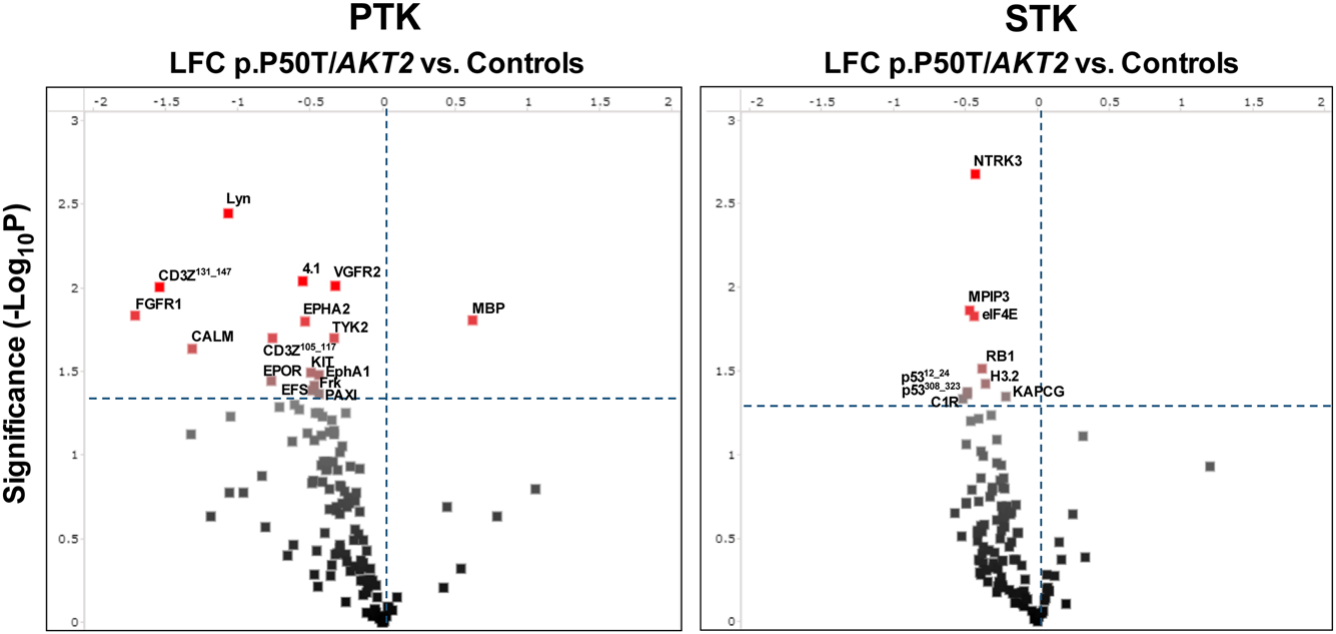
The volcano plots of differentially phosphorylated PTK and STK peptides in arrays exposed to insulin-stimulated muscle cell lysates from p.P50T/*AKT2* variant carriers and controls. X-axis shows the effect size (LFC = log_2_ fold change) and Y-axis shows the significance (−log_10_*P* - value). Red spots are peptides that are significantly different in phosphorylation between the genotypes (*P*  < 0.05, unpaired *t*-test).

Primary human myotubes from p.P50T/*AKT2* variant carriers (*n* = 9) and controls (*n* = 8) were stimulated with 100 nmol/L insulin and samples were processed for kinome profiling. QC passed signals were detected for 125 out of the total 196 peptide substrates of PTKs and for 120 out of the total 144 peptide substrates (hereinafter referred to as ‘peptides’) of STKs. These phosphorylated PTK and STK peptides, which were detected when the arrays were exposed to insulin-stimulated muscle cell lysates, are shown in heatmaps (Fig. 3). Phosphorylation of 15 PTK peptides and 9 STK peptides in the arrays was significantly decreased in the variant carriers compared to the controls, as shown in the volcano plots (Fig. 4) and Supplementary Tables 1 and 2. PTK peptides with reduced phosphorylation included calcium (Ca^2+^)-modulated protein calmodulin (CaM) and non-receptor tyrosine kinase LYN. Examples of the STK peptides with the most reduced phosphorylation included the tumour suppressor p53 and eukaryotic translation initiation factor 4E (eIF4E).

### Identification of predicted upstream kinases

**Figure 5:**
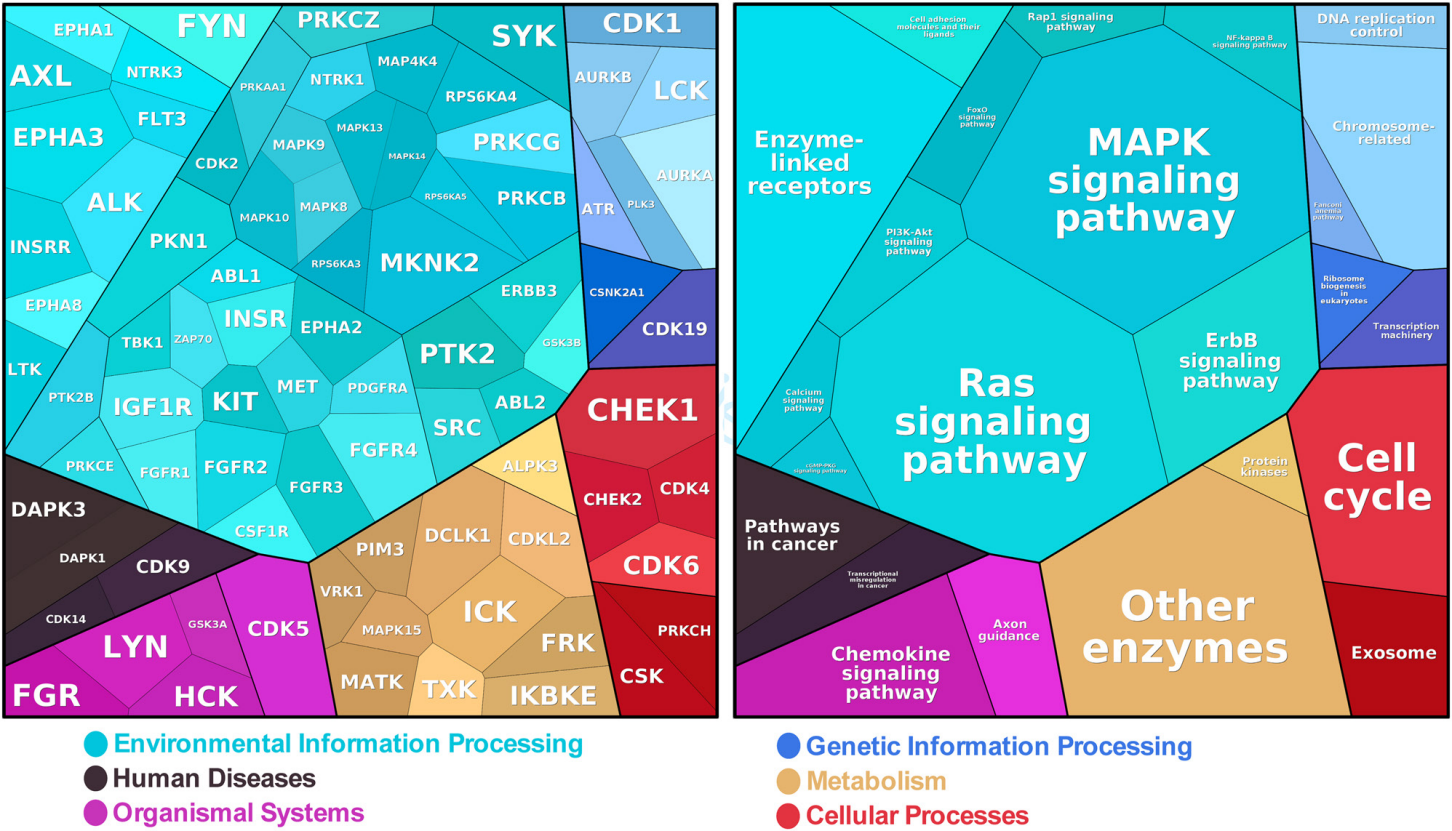
Proteomap illustrating the predicted upstream kinases (combined PTKs and STKs) (left panel) and the corresponding functional protein categories (KEGG pathway gene classification) (right panel). Each protein is shown by a polygon and functionally related proteins are arranged in common regions. The map represents the UniProt ID-derived gene name of the kinase. Left panel shows kinases with reduced activity in p.P50T/*AKT2* variant carriers. Area size of each polygon represents the median final score in p.P50T/*AKT2* variant carriers compared with the controls.

**Figure 6:**
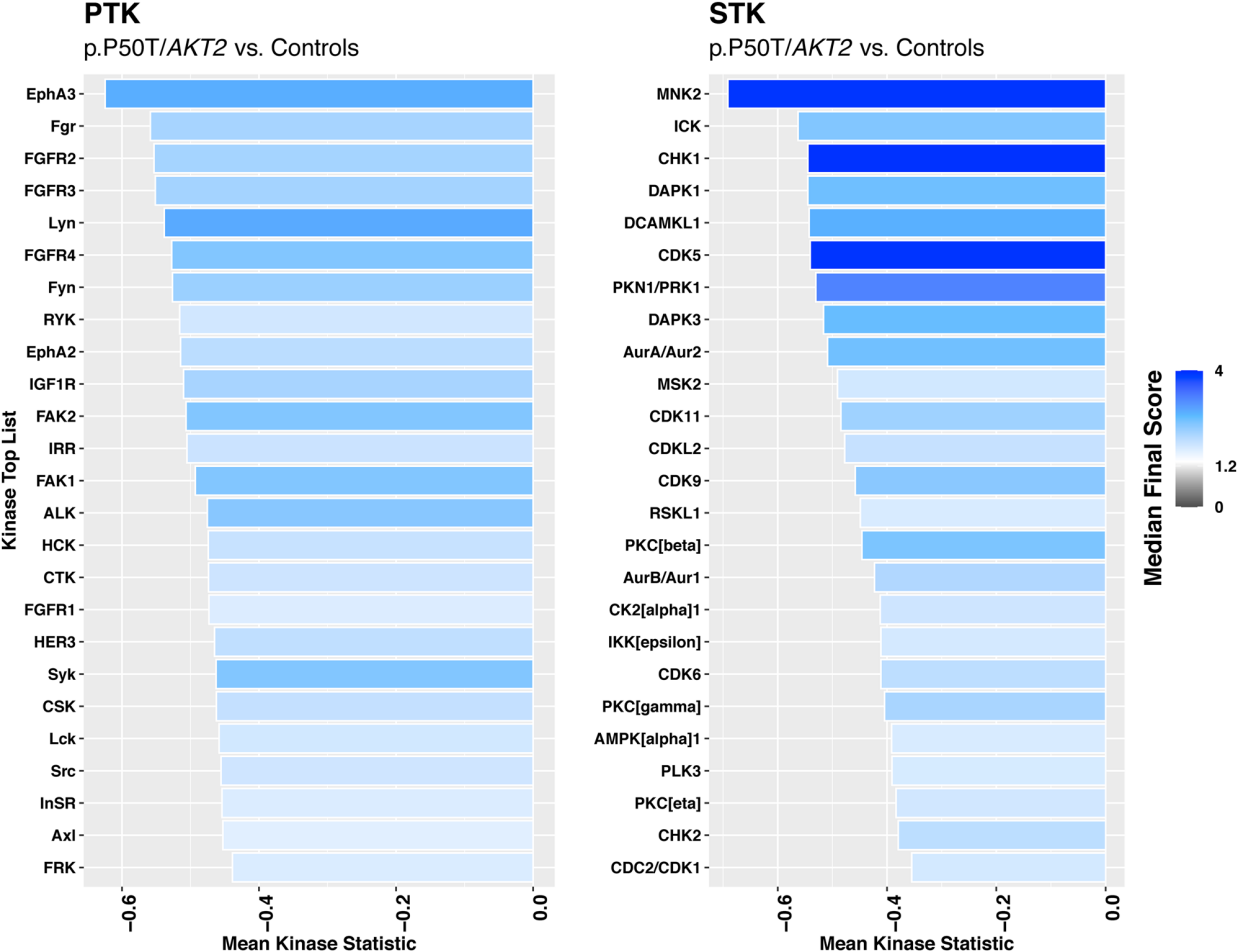
Kinase score plots of predicted upstream PTK and STK kinases responsible for the phosphorylation of peptides on PamChips. The bar graph shows the top 25 kinases (Y-axis) differentially regulated between p.P50T/*AKT2* variant carriers and controls, ranked by the median final score (a combination of specificity and significance scores). The mean kinase statistic (X-axis) indicates the mean group differences for each peptide set, with effect size (values) and direction (− = reduced activity in p.P50T/*AKT2* variant carriers).

**Figure 7:**
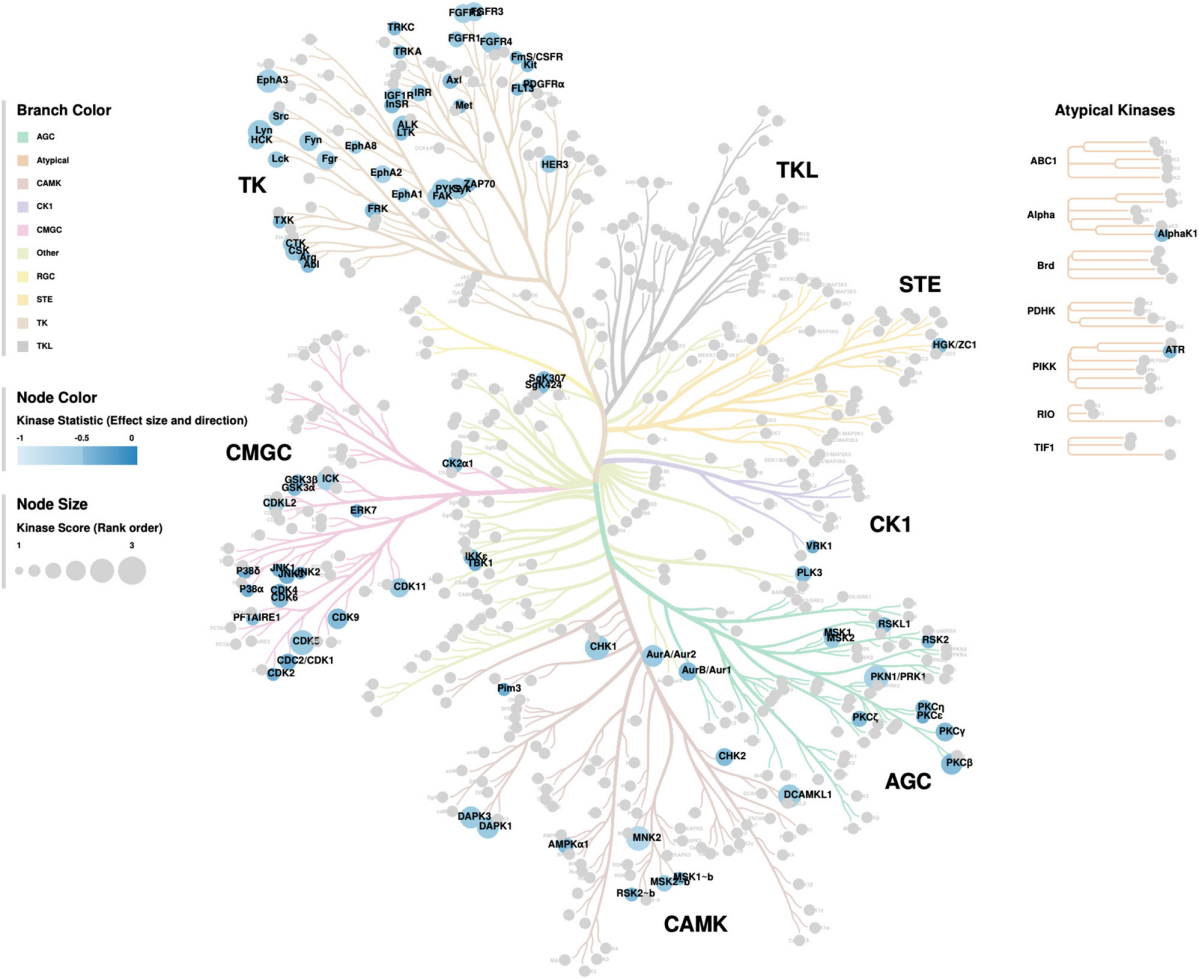
Kinome tree annotation created by Coral Kinome Tree tool. The predicted kinases (median final score >1.2) with decreased activity in p.P50T/*AKT2* variant carriers are mapped with Coral Kinome Tree tool. Colour is based on kinase statistics (median kinase statistic), and blue colour indicates decreased activity. The size of the circle is based on median final score, and larger circle size indicates higher median final score. AGC, protein kinase A (cAMP-dependent), protein kinase G (cGMP-dependent) and protein kinase C related; CAMK, Ca2+/calmodulin-dependent protein kinases; CK1, casein kinase 1; CMGC, Cdk/MAPK/GSK/Cdk-like related; RGC, receptor guanylyl cyclase; STE, Ste20, Ste11 and Ste7 related; TK, tyrosine kinases; TKL, tyrosine kinase-like.

Predicted upstream kinases with altered activity were identified using the UKA. Our analysis revealed multiple putative kinases responsible for the impaired phosphorylation of PTK and STK peptides in p.P50T/*AKT2* variant carriers. The affected upstream kinases and the corresponding pathways are illustrated in the Proteomap (Fig. 5). The top 25 of these upstream kinases are shown in the kinase score plots (Fig. 6), and the full kinase lists are presented in Supplementary Tables 3 and 4. Many affected kinases are a part of RAS and MAPK signalling pathways. The full predictive kinase data were alternatively annotated using the Coral Kinome Tree tool (Metz *et al.* 2018) to generate an intuitive kinome tree visualization of the putative kinases classified by their sequence families (Fig. 7). Our analysis shows that the putatively downregulated kinases in the p.P50T/*AKT2* variant carriers mostly belong to protein kinase families of tyrosine kinases (TK), Cdk/MAPK/GSK/Cdk-like kinases (CMGC), Ca^2+^/CaM-dependent protein kinases (CAMK) or family of protein kinase A (cAMP-dependent), protein kinase G (cGMP-dependent) and protein kinase C (AGC).

Examples of predicted upstream PTKs, with reduced activation in variant carriers, include many non-receptor SRC family tyrosine kinases, such as LYN and FGR as well as receptor tyrosine kinases, such as insulin receptor. Most affected STKs include the mitogen-activated protein kinase-interacting kinase 2 (MNK2), as well as cell cycle regulators, such as checkpoint kinase 1 (CHK1) and cyclin-dependent kinase 5 (CDK5). Other dysregulated STKs are cellular energy sensor AMP-activated protein kinase (AMPKα1) which was predicted to have reduced activity in variant carriers.

### MNK2 and FAK signalling

**Figure 8:**
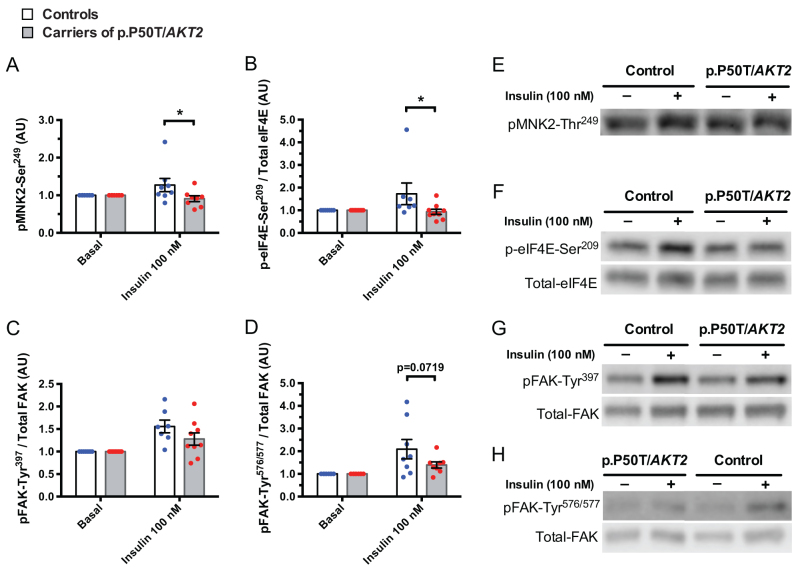
MNK2 and FAK signalling. Upstream kinase analysis suggested reduced activation of MNK2 and FAK (Fig. 6). To verify this, primary human myotubes from p.P50T/*AKT2* carriers (*n* = 8 for phosphorylated (p) MNK2-Ser^249^ and pFAK-Tyr^576^^/^^577^ and *n* = 9 for peIF4E-Ser^209^ and pFAK-Tyr^397^) and controls (*n* = 8 for pMNK2-Ser^249^ and pFAK-Tyr^576/577^ and *n* = 7 for peIF4E-Ser^209^ and pFAK-Tyr^397^) were serum-starved for 2 h and stimulated with 0 and 100 nmol/L insulin, followed by Western blot analysis. Quantification of phosphorylated (p) MNK2-Ser^249^ (A) was corrected for total lane protein detected from the PVDF membrane before antibody probing (stain-free total protein normalization-method). Quantification of p-eIF4E-Ser^209^ (B), and pFAK targets pFAK-Tyr^397^ (C) and pFAK-Tyr^576/577^ (D) were normalized to their respective total proteins. Data were normalized to the basal control sample of each subject. Representative blots are shown in (E, F, G, H). Data are expressed as mean ± s.e.m. *P < 0.05 vs control subjects, 2-way ANOVA, Sidak’s* post hoc* test. AU, arbitrary units. Open bars and blue circles = controls, light grey bars and red circles = carriers of p.P50T/*AKT2*.

To validate the findings from the bioinformatic analysis (UKA), we analysed the phosphorylation of the most affected STK – MNK2 and its downstream target eIF4E by Western blotting. Insulin-stimulated phosphorylation of MNK2 was reduced in carriers (Fig. 8A, *P* = 0.0250). Insulin-stimulated phosphorylation of eIF4E was significantly reduced in myotubes from p.P50T/*AKT2* carriers (Fig. 8B, *P* = 0.0316). Insulin-stimulated phosphorylation of PTK signalling target FAK tended to be reduced, but this did not reach statistical significance (Fig. 8C and D).

## Discussion

The aetiology of type 2 diabetes is highly complex, as there is an abundance of gene variants contributing to disease risk (Mahajan *et al.* 2018, Cai *et al.* 2020, Vujkovic *et al.* 2020). While most of the identified variants affect insulin secretion, there are also variants that contribute to insulin resistance (Manning *et al.* 2012, 2017). One example of an insulin resistance-associated gene is *AKT* which encodes one of the most important protein kinases in the insulin signalling pathway. The major isoform, AKT2, is essential for insulin action on glucose metabolism in human myotubes (Bouzakri *et al.* 2006). Studies on rare *AKT2* variants have highlighted the role of AKT2 in metabolic regulation. Inactivating *AKT2* variant leads to severe insulin resistance and lipodystrophy (George *et al.* 2004), whereas activating *AKT2* variants lead to hypoglycemia (Hussain *et al.* 2011, Arya *et al.* 2014). A partial loss-of-function *AKT2* variant, p.P50T/*AKT2*, was identified specifically in people of Finnish origin, with MAF of 1.1%. p.P50T/*AKT2* is associated with higher plasma insulin concentrations, decreased insulin sensitivity and increased risk for type 2 diabetes (Manning *et al.* 2017, Latva-Rasku *et al.* 2018). Here, we established primary muscle cell cultures from men with the p.P50T/*AKT2* variant. We observed several impairments in intracellular insulin signalling and a large-scale impairment in activities of multiple upstream tyrosine and serine-threonine kinases in primary myotubes from variant carriers.

### Glucose metabolism

Insulin increased glucose uptake and led to a dose-dependent increase in glycogen synthesis, with no difference between the ten variant carriers and eight controls. This was an unexpected result, as insulin-stimulated glucose uptake in skeletal muscle, measured with [^18^F]-FDG-PET scan *in vivo* (Latva-Rasku *et al.* 2018), was impaired in the variant carriers used in the insulin-dose response experiment (Table 1). However, these observations reflect those of Krützfeldt *et al.* who found that although insulin sensitivity was markedly different *in vivo*, there were no differences in insulin signalling or insulin action on glucose metabolism *in vitro* in cultured myotubes from insulin-sensitive and insulin-resistant people (Krützfeldt *et al.* 2000). In contrast, we have established muscle cell cultures from a total of 14 variant carriers and 14 noncarriers, with no significant difference in insulin-stimulated skeletal muscle glucose uptake *in vivo* between the genotypes (Table 1). This is in agreement with our *in vitro* data on glycogen synthesis for the whole cohort (Fig. 1C). Nevertheless, carriers in our study had higher fasting insulin concentrations and higher HOMA-IR, and the insulin-stimulated glycolysis was decreased in myotubes from p.P50T/*AKT2* variant carriers *in vitro,* suggesting an insulin-resistant phenotype.

### Insulin signalling

As AKT is a central effector in the intracellular insulin signalling network, we investigated the effect of *AKT2* gene variant on insulin signalling. Insulin stimulation led to an expected increase in phosphorylation of AKT and its downstream targets AS160 and GSK3β in primary human myotubes. Interestingly, insulin-stimulated phosphorylation of AKT-Thr^308^, GSK3β-Ser^9^ and AS160-Thr^642^ was impaired in myotubes from p.P50T/*AKT2* variant carriers. This result is in agreement with studies in HeLa cells transfected with p.P50T/AKT2 which showed reduced phosphorylation of AKT2 and a reduced ability of the AKT2 variant to phosphorylate its downstream target GSK3β (Manning *et al.* 2017). The observed defect in insulin signalling and lack of a difference in insulin action on glucose metabolism in myotubes with p.P50T/*AKT2* variant would seem contradictory. However, as p.P50T/*AKT2* is a partial loss of function variant, it is possible that its effect on glucose metabolism can be compensated by other cellular mechanisms.

### Cell proliferation

The proliferation of primary myoblasts was not affected by the presence of p.P50T/*AKT2* variant in our study. This is in agreement with an earlier study, which demonstrated that AKT1, but not AKT2, is required for cell proliferation in C2.7 mouse myoblasts (Héron-Milhavet *et al.* 2006).

### PI(3,4,5)P_3_ binding

The Pro50 residue is specific for the AKT2 isoform and lies within the lipid-binding PH domain at the protein amino terminus. Three-dimensional models of AKT2 predict a change in the conformations of the PH domain in the P50T variant, which has been hypothesized to result in inefficient recruitment of AKT2 to the plasma membrane and, thus, impaired activation of AKT2 (Manning *et al.* 2017). Phosphatidylinositol 3-kinase (PI3K)-dependent recruitment of AKT to the plasma membrane is mediated by the binding of AKT´s PH domain to PI(3,4,5)P_3_. This is followed by the phosphorylation of Ser^473^ in the C-terminal regulatory domain of AKT by the mammalian target of rapamycin complex 2 and phosphorylation of Thr^308^ in the catalytic domain by phosphoinositide-dependent kinase 1 (PDK1) (Sarbassov *et al.* 2005, Agamasu *et al.* 2017). Any defect or impairment in these events can lead to defective signalling downstream of AKT. We observed reduced binding of variant p.P50T/AKT2-PH domain to PI(3,4,5)P_3_, suggesting that recruitment of AKT2 variant to the plasma membrane is impaired and contributes to its observed partial loss-of-function characteristics (Manning *et al.* 2017).

### Analysis of kinase activities

In addition to the insulin signalling pathway, AKT is an important component of several other signalling networks. Therefore, we analysed if there were defects in the global kinome profile of myotubes from p.P50T/*AKT2* variant carriers and controls, by utilizing the PamGene^®^ kinome array technology. We found that the global kinase networks were dysregulated in the insulin-stimulated myotubes of variant carriers, as there was significantly dysregulated phosphorylation in 25 peptides. Further *in silico* analysis of the putative upstream kinases most likely driving the observed dysregulated peptide phosphorylations included >170 kinases, of which 39 PTKs and 47 STKs reached the threshold of a median final score >1.2. Mapping these kinases into a phylogenetic kinome tree showed that they belong mainly to TK, CMGC, CAMK, or AGC protein kinase families (Fig. 7).

### Protein tyrosine kinases

Prominent upstream PTKs, with downregulated activity in p.P50T/*AKT2*, included many non-receptor SRC-family kinases (SFK), such as LYN, SYK, FYN, FGR, HCK, SRC, LCK, and FRK. SFKs regulate immune response, cytoskeletal remodelling, cell survival and proliferation by acting as transducers between many cell surface receptors and intracellular signalling machinery (Parsons & Parsons 2004). One of the prominent SFKs was LYN (LCK/YES-related novel tyrosine kinase), whose autophosphorylation at residue Y397 is suggested to modulate both its kinase activity and its interaction with other phosphotyrosine-containing molecules (Sotirellis *et al.* 1995). Moreover, LYN-Y397 is phosphorylated by receptor tyrosine kinase AXL (Kimani *et al.* 2016). In our study, both phosphorylation of LYN peptide containing residue Y397 and predicted activities of LYN and AXL were decreased. These data suggest impairments in LYN activation in myotubes of p.P50T/*AKT2* variant carriers. LYN has mainly been linked to B-cell function and immune responses (Gauld & Cambier 2004). However, LYN kinase activator MLR-1023 elicits an improvement in glucose homeostasis in diabetic mice by a suggested mechanism of LYN promoting the tyrosine phosphorylation of insulin receptor substrate 1 (IRS-1), which may amplify and prolong the insulin signalling response (Ochman *et al.* 2012).

SFKs participate in the generation of Ca^2+^ signal, and, reciprocally, the Ca^2+^ signal modulates the activity of SFKs (Anguita & Villalobo 2017). CaM is a ubiquitous Ca^2+^-binding protein, whose diversely phosphorylated and non-phosphorylated forms, in the presence or absence of Ca^2+^, differentially regulate the function of hundreds of enzyme and non-enzyme proteins (Benaim & Villalobo 2002, Anguita & Villalobo 2017). CaM peptide, containing the residue Y100 (also referred Y99 in the literature), was less phosphorylated in the arrays exposed to insulin-stimulated muscle cell lysates from p.P50T/*AKT2* variant carriers. CaM-Y99 is phosphorylated by SFKs including SRC, FYN and FGR (Anguita & Villalobo 2017). Another putative downregulated PTK was the insulin receptor (InsR), which also participates in the generation of Ca^2+^ signal (Anguita & Villalobo 2017) and phosphorylates CaM-Y99 in multiple tissues (Benaim & Villalobo 2002). In endothelial cells, phosphorylated CaM-Y99 binds to the 85 kDa regulatory subunit of PI3K (PI3K-p85), and the subsequent increase of PIP_3_ permits the anchoring of nonselective, Ca^2+^-permeable cation channel, TRPC6 (transient receptor potential canonical protein 6), to the plasma membrane (Chaudhuri *et al.* 2016). Many studies show the multifaceted interplay of CaM in a variety of intracellular processes. The CaM-binding domain of AS160 regulates contraction-dependent glucose uptake in skeletal muscle (Kramer *et al.* 2007). CaM also has an effect on insulin-dependent GLUT4 translocation and glucose uptake in adipose cells (Yang *et al.* 2000, Whitehead *et al.* 2001). In addition, CaM has been suggested to bind to the AKT-PH domain in the cytoplasm, mediating AKT translocation to the plasma membrane (Agamasu *et al.* 2017). These data suggest an impaired activation of non-receptor (SFKs) and receptor (InsR) tyrosine kinases as well as CaM, which may lead to alterations in intracellular Ca^2+^ fluxes, and thus, contribute to changes in cellular functions in the muscle cells of p.P50T/*AKT2* variant carriers.

### Serine-threonine kinases

The most prominent dysregulated, *in silico* predicted upstream STK, was MAPK-interacting serine/threonine-protein kinase 2 (MNK2). MNK2 is a well-known kinase directly phosphorylating the translation initiation factor eIF4E at the residue S209. eIF4E is upregulated in a number of human cancers, and eIF4E phosphorylation by MNKs may play an important role in cancer biology (Joshi & Platanias 2014). In our study, PamGene^®^ profiling revealed that the phosphorylation of the peptide sequence of eIF4E which includes S209, as well as the predicted activity of the responsible upstream kinase MNK2, to be significantly reduced in p.P50T/*AKT2* variant carriers. This was also validated by Western blotting which demonstrated reduced insulin-stimulated phosphorylation of both MNK2-Ser^249^ and eIF4E-Ser^209^. MNK2 is suggested to suppress the expression of GLUT4, and MNK2-KO mice are markedly protected against high fat diet-induced insulin resistance and glucose intolerance (Moore *et al.* 2016). This raises the possibility that the reduction in MNK2 activity in variant carriers may enhance insulin sensitivity and contribute to the rather surprising finding of normal glucose uptake and glycogen synthesis observed in primary muscle cells of p.P50T/*AKT2* variant carriers.

Other predicted dysregulated upstream STKs, checkpoint kinase 1 (CHK1) and cyclin-dependent kinase 5 (CDK5), play roles in cell cycle regulation, and their functional aberrations have been linked to diseases such as cancers (Kastan & Bartek 2004, Sharma & Sicinski 2020). In high-glucose conditions, CHK1-mediated DNA damage response is not activated properly (Zhong *et al.* 2018), which may be associated with elevated cancer rates in people with diabetes. CHK1, as well as CDK5, phosphorylate tumour suppressor antigen p53, at least on residues S20 and S15, respectively (Shieh *et al.* 2000, Lee *et al.* 2007). The peptide sequence of p53 containing these phosphosites was significantly less phosphorylated in arrays exposed to muscle cell lysates from p.P50T/*AKT2* variant carriers, suggesting possible impaired DNA damage response and impairments in cell cycle regulation in variant carriers. p53 is a transcription factor, which is mutated in a number of cancers leading to impaired induction of cell cycle arrest, senescence and apoptosis. In addition, there is increasing evidence that p53 also plays a significant role in regulating glucose homeostasis and impacts metabolic diseases and diabetes (Kung & Murphy 2016, Itahana & Itahana 2018). The role of p53 in energy metabolism also emerges in the activation of the AMPK/p53 axis. Activation of metabolic energy sensor AMPK promotes phosphorylation of p53 at residue S15. This phosphorylation is required to initiate AMPK-dependent cell-cycle arrest; thus, AMPK-induced p53 activation promotes cellular survival in response to glucose deprivation (Jones *et al.* 2005). In our study, UKA revealed a decrease in AMPK(α1) activation.

There are also some limitations. Insulin action on glucose uptake or glycogen synthesis was not affected by the p.P50T/*AKT2* gene variant *in vitro*. While this would seem contradictory to *in vivo* situation (Latva-Rasku *et al.* 2018), dissociation between insulin action *in vivo* and *in vitro* in cultured human myotubes has previously been observed (Krützfeldt *et al.* 2000). In addition, mice lacking AKT2 in skeletal muscle displayed normal skeletal muscle insulin signalling, glucose tolerance and insulin sensitivity despite a reduction in phosphorylated AKT (Jaiswal *et al.* 2019). These data suggest that impairment in one signalling target may not be sufficient to induce insulin resistance, as other signalling pathways may compensate. As p.P50T/*AKT2* gene variant leads to a partial loss of the AKT2 function (Manning *et al.* 2017), it is possible that a gene–environment interaction is necessary for insulin resistance to fully manifest *in vivo*. Since exposure to the saturated fatty acid palmitate leads to insulin resistance (Skrobuk *et al.* 2012), we exposed primary myotubes to palmitate. This led to a reduction in insulin-stimulated glucose incorporation into glycogen, with no difference between the genotypes. As the duration of palmitate exposure was relatively short in our study, it would be interesting in the future to explore how myotubes from variant carriers respond to an overload of different nutrients in a chronic setting.

## Conclusion

Collectively, the results of the kinome profiling underline the highly interconnected pathways and untraditional players impacted by the presence of p.P50T/*AKT2* variant. The observed signalling impairments were associated with impaired *in vitro* insulin-stimulated glycolysis, fasting hyperinsulinemia and reduced insulin-stimulated skeletal muscle glucose uptake *in vivo*, and thus are likely to contribute to the insulin resistant phenotype of the carriers of p.P50T/*AKT2* variant (Latva-Rasku *et al.* 2018).

## Supplementary Material

Supplemental Material

## Declaration of interest

Dr. Savithri Rangarajan is an employee of the PamGene International B.V., s-Hertogenbosch, The Netherlands. Other authors declare no conflict of interest.

## Funding

The authors acknowledge the following funding sources: Diabetes Wellness Sverige (grant no 598- 174, (HAK)), Diabetes Wellness Finland (VMO), Finnish Cultural Foundation (HAK), Finnish Diabetes Research Foundation (HAK, VMO), Finska Läkaresällskapet (HAK), Helsinki University Hospital (funding from hospital administration and VATR (governmental subsidy for research) grants TYH2017129, TYH2018110, TYH2019223, TYH2021317) (HAK), Jalmari and Rauha Ahokas Foundation (HAK), Laboratoriolääketieteen edistämissäätiö sr (HAK), and Liv och Hälsa Foundation (HAK), and Centre of Excellence of Cardiovascular and Metabolic Diseases supported by the Academy of Finland (PN, ML). SM has received support from Doctoral School of Health Sciences (Doctoral Programme in Clinical Research) of University of Helsinki, Finnish Diabetes Research Foundation, Finska Läkaresällskapet and Suomalais-Norjalainen Lääketieteen Säätiö. The study funders were not involved in the design of the study; the collection, analysis, and interpretation of data; writing the report; and did not impose any restrictions regarding the publication of the report.

## Prior Presentation information

The results of this article have been presented as oral presentations in the European Association for the Study of Diabetes (EASD) 56th Annual Virtual Meeting, 21–25 September 2020, and in the EASD 58th Annual Meeting in Stockholm, 20–23 September 2022.

## Author contribution statement

SM, ND, YHN, ML and HAK designed the study. HAK and AL-R collected the muscle biopsies. SM, ND, SR, YHN, AL-R, PN, VMO and HAK acquired the data. SM, ND, SR, VMO and HAK analysed and interpreted the data. SM, ND, and HAK drafted the article, which was reviewed and edited by SR, YHN, AL-R, PN, VMO and ML. HAK supervised the study and acquired the funding. HAK is the guarantor of this work and, as such, had full access to all the data in the study and takes responsibility for the integrity of the data and the accuracy of the data analysis.
