# Supplementary material for: Finnish-specific AKT2 gene variant leads to impaired insulin signalling in myotubes
Source: J Mol Endocrinol. 2022 Jan 4;70(2):e210285. doi: 10.1530/JME-21-0285 (PMC9874976; doi:10.1530/JME-21-0285)
Supplement: Supplemental Material [file supplementary_material.pdf]

Online supplemental material

Supplemental Materials and Methods

Antibodies and materials

Culture medias: The culture medias used for muscle cell proliferation and differentiation were high glucose DMEM/F12 (3150 mg/L, 17.5 mmol/l, Gibco cat. 31331, Grand Island, NY, USA) and low glucose DMEM/F12 (1000 mg/L, 5.6 mmol/l, Gibco cat. 21885), respectively. Culture media used for palmitate pre-treatments and serum starvation was low glucose DMEM (Sigma cat. D5546, St. Louis, MO, USA). Culture media for glucose uptake assay was glucose-free DMEM (Gibco cat. 11966).

Antibodies: pAKT-Ser^{473} (cat. #9271), pAKT-Thr^{308} (cat. #9275**)**, total AKT (cat. #9272), pAS160-Thr^{642} (cat. #4288), total AS160 (cat. #2447), pGSK3β-Ser^{9} (cat. #9336), total GSK3β (cat. #9315), p-eIF4E-Ser^{209} (cat. #9741), total eIF4E (cat. #2067), p-FAK-Tyr^{397} (cat. #8556T), p-FAK-Tyr$^{576/577}$ (cat. #3281) and GST (cat. #2624) were from Cell Signalling Technology (Leiden, NL). pMNK2-Thr^{249} (cat. #PA5-105902) and total FAK (cat. #AHO0502) were from Invitrogen (ThermoFisher Scientific, Waltham, MA, USA). Anti-rabbit and anti-mouse HRP-conjugated secondary antibody was from Jackson ImmunoResearch Laboratories Inc. (Cambridgeshire, UK).

PamChip® reagents: M-PER (Mammalian Protein Extraction Buffer) lysis buffer and Halt Protease and Phosphatase Inhibitor Cocktail were from ThermoFisher Scientific™ (Darmstadt, DE).

Seahorse reagents: XF Glycolytic Rate Assay kit, XF DMEM medium pH 7.4, XF 1.0 mol/l Glucose Solution, XF 100 mmol/l Pyruvate Solution, and XF 200 mmol/l Glutamine Solution were from Agilent Technologies Inc. (Santa Clara, CA, USA).

Other reagents: Amphotericin B was from Gibco. Penicillin-streptomycin solution, FBS, L-glutamine, palmitic acid, L-carnitine, rat liver glycogen carrier, cytochalasin B and L-Glutathione were from Sigma. WST-1 assay kit was from Takara Bio Inc. (Shiga, JP). Fatty acid-free BSA was from Biowest (Nuaillé, FR). Actrapid Insulin was from Novo Nordisk (Bagsværd, DK). 2-[1,2-^{3}H]-deoxy-D-glucose and D-[^{14}C]-glucose were from Perkin Elmer (Boston, MO, USA). cOmplete™ EDTA-free Protease Inhibitor Cocktail tablets and PhosSTOP™ phosphatase inhibitor tablets were from Roche (Mannheim, DE). Pierce BCA Protein Assay kit, ECL2 Western Blotting Substrate kit and PIP Strips™ Membranes were from ThermoFisher Scientific (Waltham, MA, USA). PVDF membranes and chromatography columns were from Bio-Rad (Hercules, CA, USA). Glutathione Sepharose™4B was from GE Healthcare (Danderyd, SE). pGEX-4T-1 vector was from GenScript (Piscataway, NJ, USA), Rosetta DE3 competent E. Coli cells were from Novagen, MilliporeSigma (Burlington, MA, USA).

Glucose metabolism

Glucose uptake and glucose incorporation into glycogen were measured in differentiated myotubes with radioactive glucose tracers, and the glycolysis was measured with a Seahorse XF^{e}96 flux analyzer, as described (Skrobuk et al., 2012, Mäkinen et al., 2020). In brief, ***glucose uptake*** was detected in triplicate by measuring intracellular accumulation of 2-[1,2-^{3}H]-deoxy-D-glucose (final specific activity 100 mCi/mmol). Myotubes were serum-starved for 2 h and stimulated with or without insulin for 1 h at +37°C. Cytochalasin B (50 μmol/l) was used to subtract the non-specific glucose uptake. Cells were lysed and radioactivity was measured with a scintillation counter. Values were adjusted to protein concentration measured with a Pierce BCA Protein Assay kit. Data (in pmol/mg protein/min) were normalized to the basal control sample of each subject.

Glycogen synthesis was measured in triplicate by detecting D-[^{14}C]-glucose (final specific activity 0.18 µCi/µmol) incorporation into glycogen. Briefly, serum-starved or palmitate-treated myotubes were stimulated with or without insulin together with radioactive glucose tracer for 90 min at +37°C. The cells were lysed, glycogen was extracted and precipitated. Precipitated glycogen was resolved in purified H_2O and radioactivity was detected with a scintillation counter. Values were adjusted to protein concentration measured with the BCA assay. Data (in nmol/g protein/h) were normalized to the basal control sample of each subject.

Glycolytic rate was determined by measuring extracellular proton efflux rate (PER) with a Seahorse XF^{e}96 analyzer (Seahorse Bioscience, a part of Agilent Technologies, CA, USA) using the XF Glycolytic Rate Assay kit, which provides measurement of accurate glycolytic rate, excluding the contribution of mitochondrial/TCA cycle derived CO_2 to extracellular acidification. Briefly, 15 000 cells/well were plated on XF96-well microplates 8 days before the assay, and the differentiation was initiated on the next day. After 6 days of differentiation, the cells were serum-starved for 16-18 h. On the day of the assay, starvation media was replaced with XF DMEM media supplemented with 10 mmol/l glucose, 2 mmol/l glutamine, and 1 mmol/l pyruvate with or without 100 nmol/l insulin. Baseline glycolytic rate was first measured prior to any assay injections. Then, 0.5 µM Rotenone/Antimycin A (inhibitors of mitochondrial oxidative phosphorylation) and 50 mM 2-DG (inhibitor of glycolysis) were injected sequentially to measure the compensatory glycolysis and post-2-DG acidification, respectively. Values were normalized to protein concentration measured with the BCA assay. Data were normalized to the basal control of each subject.

Intracellular signalling targets were investigated in differentiated myotubes with western blotting (Skrobuk et al., 2012, Mäkinen et al., 2020). Differentiated myotubes were serum-starved for 2 h and stimulated with insulin for 10 min at +37°C. Cells were lysed in NP40-lysis buffer with

adjusted pH 7.7 (10 mmol/l TRIS, 150 mmol/l NaCl, 7 mmol/l EDTA and 0.5% (v/v) NP-40) supplemented with cOmplete protease and PhosSTOP phosphatase inhibitors. Pierce BCA Protein Assay kit was used to determine protein concentrations. Proteins were immunoblotted under reducing conditions, blocked with 5% (w/v) milk-TBST and probed with phospho-antibodies to detect phosphorylation of AKT (Ser^{473} and Thr^{308}), AS160 (Thr^{642}), GSK3β (Ser^{9}), MNK2 (Thr^{249}), eIF4E (Ser^{209}), FAK (Tyr^{397} and $Tyr^{576/577}$). Total target proteins were detected with corresponding antibodies after treating the membranes with stripping buffer (62.5 mmol/l TRIS pH 6.8, 2% (w/v) SDS, 0.7% (v/v) β-mercaptoethanol) in a shaker for 45 min at +45°C. Primary antibodies were probed with HRP-conjugated secondary antibody, visualized by enhanced chemiluminescence and quantified using Fiji software (Schindelin et al., 2012) (for signalling targets AKT, AS160, and GSK3β) or Image Lab software (Bio-Rad) (for signalling targets MNK2, eIF4E, and FAK). Intensities of the phosphorylated proteins were normalized to the intensity of their corresponding total protein. Quantification of phosphorylated MNK2-Ser^{249} was corrected for total lane protein detected from the PVDF membrane before antibody probing (stain-free total protein normalization-method). Insulin stimulation led to a dose-dependent increase in the phosphorylation of AKT (pAKT), whereas pAKT was not detectable at the basal state in the western blot analysis. Thus, for pAKT, data were normalized to the 1 nM insulin sample of each subject. For all other targets (AS160, GSK3β, MNK2, eIF4E, and FAK), data were normalized to the basal control sample of each subject.

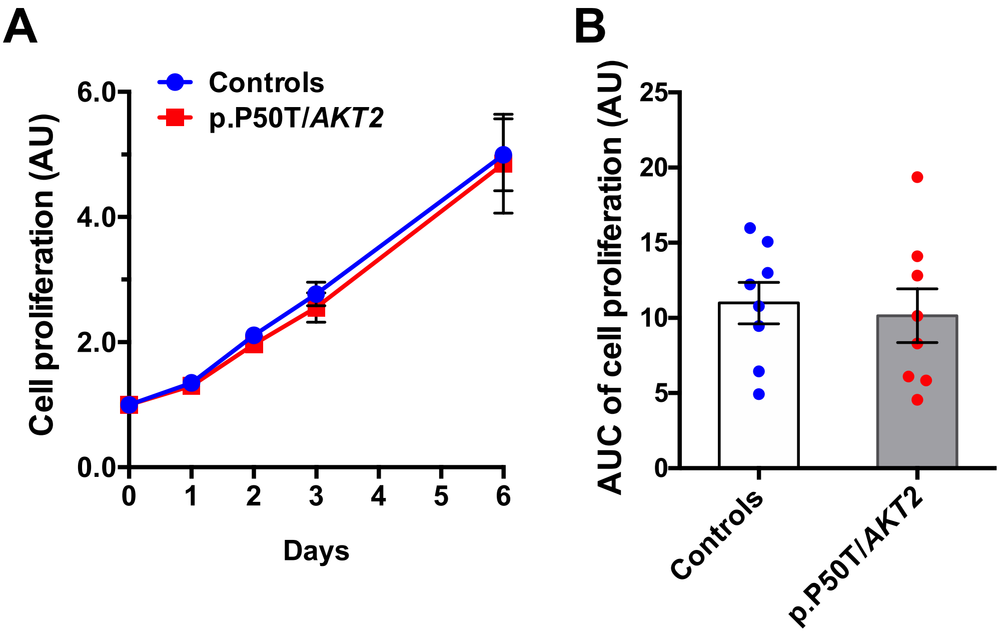

Supplemental Figure 1. Proliferation of primary human myoblasts from 8 p.P50T/*AKT2* carriers (red squares) and 8 controls (blue circles) **(A)**, measured with WST-1 assay kit. Values of each subject are normalized to their own values at timepoint "Day 0". Data are expressed as mean ± SEM. Area under the curve (AUC) is calculated to show the relative proliferation (**B**) with no difference between p.P50T/*AKT2* carriers and controls (unpaired *t*-test). Open bar and blue circles = controls, light grey bar and red circles = carriers of p.P50T/*AKT2*.

Supplemental Table 1. PTK peptides with altered phosphorylation in p.P50T/*AKT2* carriers vs. controls (p<0.05).

PTK Peptide ID (PamGene®)	UniProt ID	UniProt Accession	Peptide sequence	Log_2 fold change (carrier vs. ctrl)	p-value
LYN_391_403	Lyn	P07948	VIEDNEYTAREGA	-1.0620	0.0036
41_654_666	Protein 4.1	P11171	LDGENIYIRHSNL	-0.5504	0.0092
VGFR2_989_1001	VEGFR2	P35968	EEAPEDLYKDFLT	-0.3292	0.0097
CD3Z_135_147	CD247	P20963	KGHDGLYQGLSTA	-1.5431	0.0099
FGFR1_761_773	FGFR1	P11362	TSNQEYLDLSMPL	-1.7065	0.0147
MBP_198_210	MBP	P02686	ARTAHYGSLPQKS	0.6265	0.0156
EPHA2_765_777	EPHA2	P29317	EDDPEATYTTSGG	-0.5381	0.0159
TYK2_1048_1060	TYK2	P29597	VPEGHEYYRVRED	-0.3328	0.0199
CD3Z_105_117	CD247	P20963	NPQEGLYNELQKD	-0.7561	0.0200
CALM_95_107	Calmodulin 1	P0DP23	KDGNGYISAAELR	-1.3153	0.0230
KIT_930_942_C942S	KIT	P10721	ESTNHIYSNLANS	-0.4925	0.0323
EPHA1_774_786	EPHA1	P21709	LDDFDGTYETQGG	-0.4362	0.0335
EPOR_419_431	EPOR	P19235	ASAASFEYTILDP	-0.7654	0.0359
FRK_380_392	FRK	P42685	KVDNEDIYESRHE	-0.4700	0.0383
EFS_246_258	EFS	O43281	GGTDEGIYDVPLL	-0.4835	0.0409
PAXI_111_123	Paxillin	P49023	VGEEEHVYSFPNK	-0.4374	0.0428

Supplemental Table 1. Insulin-stimulated muscle cell lysates from carriers of p.P50T/*AKT2* and controls were subjected to a kinome analysis utilizing a protein tyrosine (PTK) peptide substrate array (PamGene® kinome profiling system). Phosphorylation of 16 PTK peptides was significantly altered; 15 peptides with decreased and one (1) peptide with increased phophorylation in p.P50T/*AKT2* variant carriers compared to controls ($p<0.05$, Student´s unpaired *t*-test). PTK Peptide ID (PamGene®) shows the numbers for indicated amino acid sequence.

Supplemental Table 2. STK peptides with reduced phosphorylation in p.P50T/*AKT2* carriers vs. controls (p<0.05).

**STK Peptide ID (PamGene®)**	**UniProt ID**	**UniProt Accession**	**Peptide sequence**	**Log_2 fold change (carrier vs. ctrl)**	**p-value**
NTRK3_824_836	NTRK3	Q16288	LHALGKATPIYLD	-0.4320	0.0021
MPIP3_208_220	CDC25	P30307	RSGLYRSPSMPEN	-0.4697	0.0138
IF4E_203_215	eIF4E	P06730	TATKSGSTTKNRF	-0.4335	0.0148
RB_242_254	RB1	P06400	AVIPINGSPRTPR	-0.3791	0.0307
H32_3_18	Histone H3.2	Q71DI3	RTKQTARKSTGGKAPR	-0.3555	0.0377
P53_12_24	TP53	P04637	PPLSQETFSDLWK	-0.4837	0.0420
P53_308_323	TP53	P04637	LPNNTSSSPQPKKKPL	-0.4882	0.0433
KAPCG_192_206	PKA C-gamma	P22612	VKGRTWTLCGTPEYL	-0.2143	0.0448
C1R_201_213	C1R	P00736	ASGYISSLEYPRS	-0.5196	0.0464

Supplemental Table 2. Insulin-stimulated muscle cell lysates from carriers of p.P50T/*AKT2* and controls were subjected to a kinome analysis utilizing a serine-threonine (STK) peptide substrate array (PamGene® kinome profiling system). Phosphorylation of 9 STK peptides was significantly reduced in p.P50T/*AKT2* variant carriers compared to controls ($p<0.05$, Student´s unpaired *t*-test). STK Peptide ID (PamGene®) shows the numbers for indicated amino acid sequence.

Supplemental Table 3. Downregulated upstream PTK kinases in p.P50T/*AKT2* carriers.

Rank	PTK kinase ID	UniProt Accession	Full kinase name(s)	Median final score	Mean kinase statistic (carrier vs. ctrl)
1	Lyn	P07948	Tyrosine-protein kinase Lyn<br>Lck/Yes-related novel protein tyrosine kinase	2.8182	-0.5383
2	EphA3	P29320	Ephrin type-A receptor 3	2.7881	-0.6246
3	FAK1 (PTK2)	Q05397	Focal adhesion kinase 1	2.4442	-0.4929
4	FGFR4	P22455	Fibroblast growth factor receptor 4	2.4437	-0.5274
5	Syk	P43405	Tyrosine-protein kinase SYK<br>Spleen tyrosine kinase	2.4379	-0.4626
6	FAK2 (PTK2B)	Q14289	Protein-tyrosine kinase 2-beta<br>Focal adhesion kinase 2<br>Calcium-dependent tyrosine kinase	2.4351	-0.5065
7	ALK	Q9UM73	ALK tyrosine kinase receptor<br>Anaplastic lymphoma kinase	2.3928	-0.4755
8	Fyn	P06241	Tyrosine-protein kinase Fyn<br>Proto-oncogene c-Fyn	2.2396	-0.5263
9	FGFR3	P22607	Fibroblast growth factor receptor 3	2.1744	-0.5514
10	FGFR2	P21802	Fibroblast growth factor receptor 2	2.1675	-0.5532
11	Fgr	P09769	Tyrosine-protein kinase Fgr<br>Proto-oncogene c-Fgr	2.1446	-0.5586
12	IGF1R	P08069	Insulin-like growth factor 1 receptor	2.1432	-0.5100
13	EphA2	P29317	Ephrin type-A receptor 2<br>Epithelial cell kinase	1.9803	-0.5145
14	HER3 (ERBB3)	P21860	Receptor tyrosine-protein kinase erbB-3<br>Proto-oncogene-like protein c-ErbB-3	1.9385	-0.4650
15	CSK	P41240	Tyrosine-protein kinase CSK<br>C-Src kinase	1.9043	-0.4622
16	HCK	P08631	Tyrosine-protein kinase HCK<br>Hematopoietic cell kinase	1.8716	-0.4741
17	IRR (INSRR)	P14616	Insulin receptor-related protein	1.8431	-0.5052
18	CTK (MATK)	P42679	Megakaryocyte-associated tyrosine-protein kinase<br>Hematopoietic consensus tyrosine-lacking kinase	1.8253	-0.4737
19	Src	P12931	Proto-oncogene tyrosine-protein kinase Src	1.8052	-0.4553
20	RYK (not presented in Proteomap)	H0Y8A4	Tyrosine-protein kinase RYK	1.7840	-0.5159
21	Lck	P06239	Tyrosine-protein kinase Lck<br>Lymphocyte cell-specific protein-tyrosine kinase	1.7809	-0.4582
22	FRK	P42685	Tyrosine-protein kinase FRK<br>FYN-related kinase	1.6991	-0.4389
23	InSR	P06213	Insulin receptor	1.6828	-0.4540
24	FGFR1	P11362	Fibroblast growth factor receptor 1	1.6761	-0.4731
25	Axl	P30530	Tyrosine-protein kinase receptor UFO<br>AXL oncogene	1.6163	-0.4527
26	EphA1	P21709	Ephrin type-A receptor 1<br>Erythropoietin-producing hepatoma receptor	1.4569	-0.4538
27	Met	P08581	Hepatocyte growth factor receptor<br>Tyrosine-protein kinase Met	1.4267	-0.4395
28	TRKC (NTRK3)	Q16288	NT-3 growth factor receptor<br>Neurotrophic tyrosine kinase receptor type 3	1.4009	-0.4525
29	ZAP70	P43403	Tyrosine-protein kinase ZAP-70<br>70 kDa zeta-chain associated protein	1.3872	-0.4241
30	Abl (ABL1)	P00519	Tyrosine-protein kinase ABL1<br>Abelson tyrosine-protein kinase 1<br>Proto-oncogene c-Abl	1.3861	-0.4208

31	TRKA (NTRK1)	P04629	High affinity nerve growth factor receptor<br>Neurotrophic tyrosine kinase receptor type 1<br>Tropomyosin-related kinase A	1.3312	-0.4234
32	EphA8	P29322	Ephrin type-A receptor 8	1.3175	-0.4589
33	FmS/CSFR	P07333	Macrophage colony-stimulating factor 1 receptor	1.3134	-0.4188
34	TXK	P42681	Tyrosine-protein kinase TXK	1.3080	-0.4128
35	Arg (ABL2)	P42684	Tyrosine-protein kinase ABL2<br>Tyrosine-protein kinase ARG<br>Abelson-related gene protein	1.3001	-0.4141
36	Kit	P10721	Mast/stem cell growth factor receptor Kit	1.2932	-0.4258
37	LTK	P29376	Leukocyte tyrosine kinase receptor	1.2878	-0.4331
38	PDGFR[alpha]	P16234	Platelet-derived growth factor receptor alpha	1.2821	-0.4504
39	FLT3	P36888	Receptor-type tyrosine-protein kinase FLT3<br>Fms-like tyrosine kinase 3	1.2246	-0.3989

Supplemental Table 3. Upstream kinase analysis based on the phosphorylated PTK peptide (substrate) array (PamGene® kinome profiling system). 39 PTKs were identified as putative upstream kinases with reduced activity in myotubes of p.P50T/*AKT2* variant carriers compared to controls, ranked by median final score (>1.2). The Proteomap ID (see Fig. 5) represents the gene name and is marked here in parenthesis if different from PTK Kinase ID.

Supplemental Table 4. Downregulated upstream STK kinases in p.P50T/*AKT2* carriers.

Rank	STK kinase ID	UniProt Accession	Full kinase name(s)	Median final score	Mean kinase statistic (carrier vs. ctrl)
1	CHK1 (CHEK1)	O14757	Serine/threonine-protein kinase Chk1<br>Checkpoint kinase 1	3.9508	-0.5450
2	CDK5	Q00535	Cyclin-dependent-like kinase 5	3.9355	-0.5406
3	MNK2 (MKNK2)	Q9HBH9	MAP kinase-interacting Ser/Thr-protein kinase 2	3.9297	-0.6913
4	PKN1/PRK1	Q16512	Ser/Thr-protein kinase N1/Protein-kinase C-related kinase 1	3.1938	-0.5304
5	DCAMKL1 (DCLK1)	O15075	Serine/threonine-protein kinase DCLK1<br>Doublecortin-like and CAM kinase-like 1	2.7607	-0.5426
6	DAPK3	O43293	Death-associated protein kinase 3	2.6007	-0.5163
7	DAPK1	P53355	Death-associated protein kinase 1	2.5654	-0.5450
8	AurA/Aur2 (AURKA)	O14965	Aurora kinase A<br>Aurora/IPL1-related kinase 1	2.5528	-0.5088
9	PKC[beta] (PRKCB)	P05771	Protein kinase C beta type	2.4810	-0.4460
10	ICK	Q9UPZ9	Serine/threonine-protein kinase ICK<br>Intestinal cell kinase	2.4471	-0.5628
11	CDK9	P50750	Cyclin-dependent kinase 9	2.3768	-0.4582
12	CDK11 (CDK19)	Q9BWU1	Cyclin-dependent kinase 19<br>Cyclin-dependent kinase 11	2.2115	-0.4844
13	PKC[gamma] (PRKCG)	P05129	Protein kinase C gamma type	2,1236	-0.4045
14	AurB/Aur1 (AURKB)	Q96GD4	Aurora kinase B<br>Aurora/IPL1-related kinase 2	2.0741	-0.4230
15	CHK2 (CHEK2)	O96017	Serine/threonine-protein kinase Chk2<br>Checkpoint kinase 2	1.9671	-0.3796
16	CDK6	Q00534	Cyclin-dependent kinase 6	1.9605	-0.4112
17	CDKL2	Q92772	Cyclin-dependent kinase-like 2	1.8785	-0.4775
18	CK2[alpha]1 (CSNK2A1)	P68400	Casein kinase II subunit alpha	1.8120	-0.4126
19	PKC[eta] (PRKCH)	P24723	Protein kinase C eta type	1.7847	-0.3835
20	MSK2 (RPS6KA4)	O75676	Ribosomal protein S6 kinase alpha-4<br>Nuclear mitogen- and stress-activated protein kinase 2	1.7735	-0.4907
21	CDC2/CDK1	P06493	Cyclin-dependent kinase 1	1.7577	-0.3547
22	IKK[epilon] (IKBKE)	Q14164	Inhibitor of nuclear factor kappa-B kinase subunit epsilon	1.7480	-0.4112
23	PLK3	Q9H4B4	Serine/threonine-protein kinase PLK3<br>Polo-like kinase 3	1.7211	-0.3910
24	AMPK[alpha]1 (PRKAA1)	Q13131	AMP-activated protein kinase catalytic subunit alpha-1	1.7172	-0.3917
25	RSKL1 (not presented in Proteomap)	Q96S38	Ribosomal protein S6 kinase delta-1	1.7151	-0.4488
26	PKC[zeta] (PRKCZ)	Q05513	Protein kinase C zeta type	1.7150	-0.3887
27	JNK3 (MAPK10)	P53779	Mitogen-activated protein kinase 10<br>c-Jun N-terminal kinase 3	1.6895	-0.3399
28	SgK307 (not presented in Proteomap)	Q8IWB6	Inactive serine/threonine-protein kinase TEX14<br>Sugen kinase 307<br>Testis-expressed sequence 14 protein	1.6678	-0.4320
29	HGK/ZC1 (MAP4K4)	O95819	Mitogen-activated protein kinase kinase kinase kinase 4<br>HPK/GCK-like kinase HGK	1.6632	-0.4064
30	AlphaK1 (ALPK3)	Q96L96	Alpha-protein kinase 3	1.5781	-0.4095
31	ATR	Q13535	Serine/threonine-protein kinase ATR<br>Ataxia telangiectasia and Rad3-related protein	1.4471	-0.3703
32	CDK4	P11802	Cyclin-dependent kinase 4	1.4368	-0.3367
33	GSK3[beta] (GSK3B)	P49841	Glycogen synthase kinase beta	1.4304	-0.3694

34	RSK2 (RPS6KA3)	P51812	Ribosomal protein S6 kinase alpha-3<br>Ribosomal S6 kinase 2	1.4027	-0.3743
35	GSK3[alpha] (GSK3A)	P49840	Glycogen synthase kinase alpha	1.3756	-0.3325
36	p38[delta] (MAPK13)	O15264	Mitogen-activated protein kinase 13<br>MAP kinase p38 delta	1.3705	-0.3241
37	PFTAIRE1 (CDK14)	O94921	Cyclin-dependent kinase 14<br>Serine/threonine-protein kinase PFTAIRE-1	1.3363	-0.3660
38	PKC[epsilon] (PRKCE)	Q02156	Protein kinase C epsilon type	1.3176	-0.3398
39	JNK1 (MAPK8)	P45983	Mitogen-activated protein kinase 8<br>c-Jun N-terminal kinase 1	1.3028	-0.3124
40	MSK1 (RPS6KA5)	O75582	Ribosomal protein S6 kinase alpha-5<br>Nuclear mitogen- and stress-activated protein kinase 1	1.2768	-0.3665
41	VRK1	Q99986	Serine/threonine-protein kinase VRK1<br>Vaccinia-related kinase 1	1.2651	-0.3310
42	Pim3	Q86V86	Serine/threonine-protein kinase pim-3	1.2649	-0.3114
43	CDK2	P24941	Cyclin-dependent kinase 2	1.2647	-0.3259
44	MAPK14	Q16539	Mitogen-activated protein kinase 14<br>MAP kinase p38 alpha	1.2477	-0.3215
45	ERK7 (MAPK15)	Q8TD08	Mitogen-activated protein kinase 15<br>Extracellular signal-regulated kinase 7	1.2434	-0.3503
46	TBK1	Q9UHD2	Serine/threonine-protein kinase TBK1<br>TANK-binding kinase 1	1.2363	-0.3237
47	JNK2 (MAPK9)	P45984	Mitogen-activated protein kinase 9<br>c-Jun N-terminal kinase 2	1.2305	-0.3020

Supplemental Table 4. Upstream kinase analysis based on the phosphorylated STK peptide (substrate) array (PamGene® kinome profiling system). 47 STKs were identified as putative upstream kinases with reduced activity in myotubes of p.P50T/*AKT2* variant carriers compared to controls, ranked by median final score (>1.2). The Proteomap ID (see Fig. 5) represents the gene name and is marked here in parenthesis if different from STK Kinase ID.

Mäkinen S, Datta N, Nguyen YH, Kyrylenko P, Laakso M & Koistinen HA 2020 Simvastatin profoundly impairs energy metabolism in primary human muscle cells. *Endocr Connect* **9** 1103-1113.

Schindelin J, Arganda-Carreras I, Frise E, Kaynig V, Longair M, Pietzsch T, Preibisch S, Rueden C, Saalfeld S, Schmid B, *et al*. 2012. Fiji: an open-source platform for biological-image analysis. *Nat Methods* **9** 676-82.

Skrobuk P, Kraemer S, Semenova MM, Zitting A & Koistinen HA 2012 Acute exposure to resveratrol inhibits AMPK activity in human skeletal muscle cells. *Diabetologia* **55** 3051-3060.
